# Integration of amorphous ferromagnetic oxides with multiferroic materials for room temperature magnetoelectric spintronics

**DOI:** 10.1038/s41598-020-58592-5

**Published:** 2020-02-27

**Authors:** Humaira Taz, Bhagwati Prasad, Yen-Lin Huang, Zuhuang Chen, Shang-Lin Hsu, Ruijuan Xu, Vishal Thakare, Tamil Selvan Sakthivel, Chenze Liu, Mark Hettick, Rupam Mukherjee, Sudipta Seal, Lane W. Martin, Ali Javey, Gerd Duscher, Ramamoorthy Ramesh, Ramki Kalyanaraman

**Affiliations:** 1 Department of Materials Science and Engineering, University of California, Berkeley, CA 94720 USA; 2 Bredesen Center, University of Tennessee, Knoxville, TN 37996 USA; 3 School of Materials Science and Engineering, Harbin Institute of Technology, Shenzhen, 518055 P. R. China; 4 Advanced Materials Processing and Analysis Center (AMPAC, Materials Science and Engineering (MSE) Department, University of Central Florida, Orlando, FL 32816 USA; 5 Department of Materials Science and Engineering, University of Tennessee, Knoxville, TN 37996 USA; 6 Department of Electrical Engineering and Computer Sciences, University of California, Berkeley California, 94720 USA; 7 Department of Physics, Lovely Professional University, Phagwara, Punjab 144411 India; 8 College of Medicine, University of Central Florida, Orlando, FL 32827 USA; 9 Materials Sciences Division, Lawrence Berkeley National Laboratory, Berkeley California, 94720 USA; 10 Department of Chemical and Biomolecular Engineering, University of Tennessee, Knoxville, TN 37996 USA

**Keywords:** Materials science, Nanoscience and technology, Physics

## Abstract

A room temperature amorphous ferromagnetic oxide semiconductor can substantially reduce the cost and complexity associated with utilizing crystalline materials for spintronic devices. We report a new material (Fe_0.66_Dy_0.24_Tb_0.1_)_3_O_7-x_ (FDTO), which shows semiconducting behavior with reasonable electrical conductivity (~500 mOhm-cm), an optical band-gap (2.4 eV), and a large enough magnetic moment (~200 emu/cc), all of which can be tuned by varying the oxygen content during deposition. Magnetoelectric devices were made by integrating ultrathin FDTO with multiferroic BiFeO_3_. A strong enhancement in the magnetic coercive field of FDTO grown on BiFeO_3_ validated a large exchange coupling between them. Additionally, FDTO served as an excellent top electrode for ferroelectric switching in BiFeO_3_ with no sign of degradation after ~10^10^ switching cycles. RT magneto-electric coupling was demonstrated by modulating the resistance states of spin-valve structures using electric fields.

## Introduction

Spintronics aims to utilize the charge and spin of electrons in order to revolutionize technologies for information storage and logic by bringing them together onto a single chip^1,2^. Current state-of-the-art in magnetoelectronics is based on using multilayers of crystalline oxides in which significant effort must be placed in achieving low defect densities through careful materials selection and processing routes^3–8^. Thin film amorphous semiconductors are excellent candidates for spintronic applications related to switching, storage, and logic for multiple reasons^9–20^. Amorphous films have the advantage that they can be deposited at room temperature, making it possible to integrate them for electronic applications with a wider spectrum of material types.

The ternary amorphous oxide system of In-Ga-Zn-O has been successfully utilized to fabricate transparent channel layers in thin film transistors for display applications^17,21–23^. These materials exhibit a suitable combination of transparency, conductivity, mobility, and carrier concentration - all properties needed for switching applications - but they do not possess room-temperature magnetism. Therefore, while control of electronic transport is on a relatively good footing, the use of amorphous semiconductors for magnetoelectric spintronics has not yet emerged. Several groups have attempted to introduce magnetism into semiconductors by doping^24^. For instance, conventional III-V semiconductors such as GaAs have been doped with magnetic cations to form dilute magnetic semiconductors. However, these materials have still not achieved room temperature ferromagnetism. On the other hand, dilute magnetic oxides have been prepared by doping transparent heavy transition-metal oxides with magnetic cations^25–27^ and these have shown to have Curie temperatures above 300 K. A few complex oxides, such as La_0.7_Sr_0.3_MnO_3_ ^28^, Sr_2_FeMoO_6_ ^29^, and Fe_3_O_4_^30,31^ display room-temperature magnetism and metallic conductivity. However, all of these magnetic systems require good crystallinity in order to preserve the room temperature magnetism and metallicity that arises from long range spin order; they lose their magnetic order when disordered or highly defective.

Magnetoelectric multiferroics provide a pathway to control the magnetic state of such devices with an applied electric field for low-power operation^2^. Recent studies have demonstrated the use of spin valves made out of conventional ferromagnets such as Co-Fe to demonstrate magnetoelectric coupling^32^. However, one concern with such an approach is the formation of strong Schottky barriers at the interface between the metal and the oxide ferroelectric; this invariably leads to degradation issues such as polarization fatigue^33–35^, imprint and in the case of ferromagnets such as CoFe, the possibility for interfacial oxidation of the Co^36^. In contrast, oxide metals such as La-Sr-Co-O3, SrRuO3 have been demonstrated to show a strong resistance to such interfacial degradation, mainly because they form Ohmic (or almost Ohmic) contacts to the ferroelectrics^33–35^. Furthermore, such oxide metals are able to accommodate the transport of charged oxygen vacancies and thus prevent the formation of interfacial charged layers that have been identified as a possible cause for the degradation. On the other hand, ferromagnetic oxides, such as La_0.7_Sr_0.3_MnO_3_ and Fe_3_O_4_, have been integrated with BiFeO_3_ for magnetoelectric switching applications but exhibit coupling only at low temperature (<100 K)^36^. Thus, there is a need for a ferromagnetic oxide that has reasonable conductivity and large enough magnetic moment at room temperature (i.e., that can be sensed from the outside environment without the need for sophisticated sensing apparatus) and is able to withstand the deleterious effects of transport of charged point defects. We note that in this regard, while a large number of crystalline oxide metals have been explored, amorphous oxides have not been that well studied. Recent reports have emerged of amorphous materials made from iron and lanthanide systems that appear to show promising electronic or magnetic behavior^37,38^ and this forms the background for our studies.

In this work, we report the synthesis, characterization, and magnetoelectric manifestation of an amorphous semiconducting and ferromagnetic oxide thin-film material grown by pulsed-laser deposition (PLD) from the Terfenol-D (Fe_1.92_Dy_0.7_Tb_0.3_) metallic system under varying oxygen background pressures ranging from 10^−7^ to 10^−3^ Torr. The films were deposited at room temperature onto fused quartz substrates or single crystalline BiFeO_3_/SrRuO_3_/SrTiO_3_(001) substrates using  PLD (for details, see Methods section). Microscopic evidence for a homogeneous amorphous microstructure was obtained using high-resolution transmission electron microscopy (TEM), while the chemical composition and oxidation state of the (Fe_0.66_Dy_0.24_Tb_0.1_)_3_O_7-x_ films was determined using electron energy loss spectroscopy (EELS) and X-ray photoelectron spectroscopy (XPS). Electrical conduction in the films occurs by variable range hopping with conductivity decreasing with increasing oxygen pressure. Room-temperature magnetic measurements using a SQUID magnetometer revealed ferromagnetic hysteresis with a magnetic moment and coercivity decreasing and increasing, respectively, with increasing oxygen pressure. X-ray magnetic circular dichroism (XMCD) identified the source of magnetism to be the Fe^2+^/Fe^3+^ cations with the total moment of the system being highly dependent on the ratio of the two valence states of iron. The pressure-dependent measurements revealed that films deposited with oxygen pressure in the range of 1–3 × 10^−6^ Torr had a promising combination of properties for spintronic applications, including a high magnetic moment (~400 emu/cc) and low resistivity (~8 mOhm-cm). We also ascertained that this amorphous oxide can be a promising magneto-electric coupling layer to BiFeO_3_ that can also be used as a contact electrode by demonstrating at least10^10^ ferroelectric switching cycles of BiFeO_3_.

## Results and Discussion

**Figure 1:**
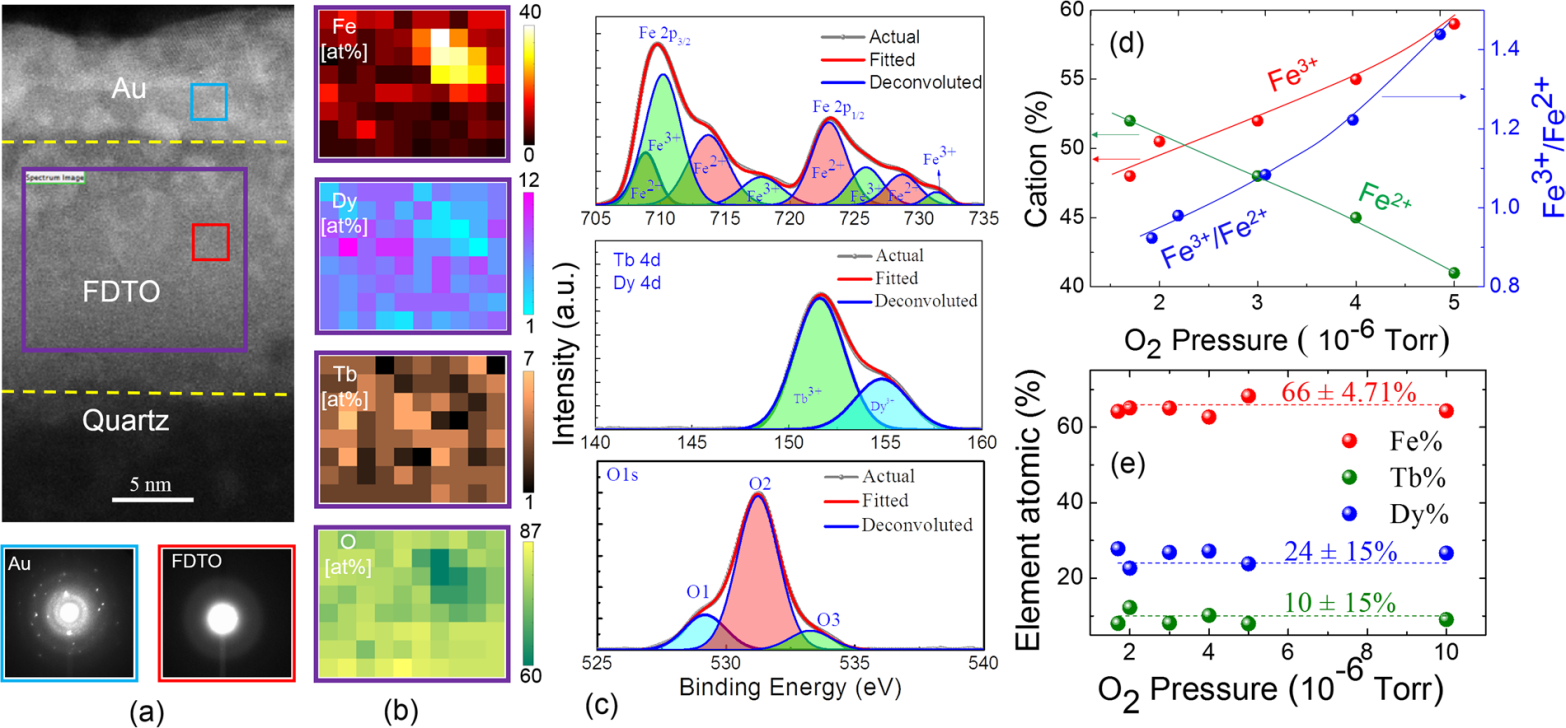
Amorphous microstructure and cation composition of FDTO. Panel (a) HRTEM image showing protective top Au layer, middle FDTO layer, and bottom quartz layer (top panel). Bottom panels shows the diffraction pattern taken from the FDTO layer (red box) and Au layer (blue box). Panel (b) The elemental distribution of Fe, Dy, Tb and O (top to bottom) obtained from EELS spectrum of the area inside the purple box. (**c**) XPS signal and peak fitting for the various metals and oxygen. (**d**) Quantification of the observed valence states of Fe (Fe^2+^ and Fe^3+^; left y-axis), and the ratio of Fe^3+^/Fe^2+^ (right y-axis) across varying oxygen pressure from 1 × 10^−6^ to 5 × 10^−6^ Torr. (**e**) Atomic % of the three metals measured by energy dispersive spectroscopy for films deposited at the various pressures, showing elemental composition of a-FDTO to be very close to that of the metallic target and consistent over all pressures. For Figure (**a**–**c**), a film deposited at 2 × 10^−6^ Torr of pressure was used.

The microstructure, compositional homogeneity and the chemical state of each of the metals in FDTO films were investigated by a combination of high-resolution imaging and electron energy loss spectroscopy (EELS) in the TEM and x-ray photoelectron spectroscopy (XPS). Figure 1(a) shows a representative FDTO film (deposited at room temperature under 2 10^−6^ Torr pressure) sandwiched between the underlying quartz substrate and a protective Au overlayer. Nano-diffraction patterns from the FDTO layer marked by the red box are shown in the bottom left panel in Fig. 1(a) and consist of a diffuse pattern that is typical of an amorphous microstructure. In contrast, the diffraction pattern from the blue box within the top Au layer showed sharp rings corresponding to a polycrystalline microstructure. Areal concentration distribution of Fe, Dy, Tb and O by EELS measured from the area marked by the purple rectangle showed that the distribution of the elements was homogeneous over the probed area, as shown in Fig. 1(b). Small regions showed higher concentrations of iron cations due to variations in the thickness of the cross-section sample causing the lanthanide peaks to be under-estimated at the expense of over-estimating the iron peak. No evidence of metal clustering or structural order was found from such TEM studies indicating that FDTO films were amorphous oxides. In order to obtain the film thicknesses and corresponding densities, the FDTO films were also characterized using X-Ray Reflectivity (XRR). There is a slight trend of the densities decreasing and thickness values increasing as oxygen deposition pressure increases for the films grown at same deposition conditions (see Fig. S1, supplementary information). Given that the deposition times were kept constant, the increase in the thickness was attributed to increasing volume with increasing oxygen pressures as a result of higher oxygen incorporation.

The oxidation state of the metal cations for films prepared under varying oxygen pressures was investigated using XPS measurements. In Fig. 1(c), the peaks obtained from iron 2p (top panel), terbium and dysprosium 4d (middle panel), and oxygen 1 s (bottom panel) for the film deposited at 2 × 10^−6^ Torr are shown. The key result was that no metallic peaks were seen from iron, terbium, and dysprosium for all the pressures investigated suggesting that the metal constituents were completely oxidized. The XPS spectra taken from all FDTO films deposited at varying oxygen pressures are shown in Fig. S2 (see supplementary information). For the entire pressure range investigated, terbium showed XPS peaks corresponding to the composition of Tb_4_O_7_, which is a mixed phase of Tb^3+^ and Tb^4+^ with excitation at 151.6 ± 0.2 eV, while dysprosium showed excitation corresponding to Dy_2_O_3_, which occurs at 154.7 ± 0.1 eV. On the other hand, the behavior of the iron cation states of Fe^3+^ to Fe^2+^ changed systematically with oxygen pressure. Using peak positions obtained from the standard XPS database, the Fe^2+^ and Fe^3+^ peaks could be decoupled thus allowing quantification of the two valence states of iron. Figure 1(d) shows the %Fe^2+^ decreasing and %Fe^3+^ increasing with increasing oxygen pressure. While the ratio Fe^3+^/Fe^2+^ was observed to increase with increasing oxygen pressure, all the films were found to be richer in Fe^2+^ compared to in Fe_3_O_4_, where the ratio of Fe^3+^/Fe^2+^ is 2. These results validated the conclusion that the thin films made by room-temperature deposition correspond to amorphous materials with completely oxidized metal constituents. The co-existence of Fe^3+^/Fe^2+^ is one of the key aspects of using an amorphous layer such as FDTO, thus enabling conduction through hopping mechanisms as well as facilitating magnetism. The cationic stoichiometry of the oxide was verified by using energy dispersive X-ray spectroscopy in an SEM to measure the metallic compositions along with summing the oxygen content according to the stoichiometry of the metal oxides identified by XPS. Figure 1(e) shows that the atomic percentage of Fe, Tb, and Dy were found to be 66 ± 10%, 10 ± 15% and 24 ± 15% respectively in the FDTO films, which is consistent with the metal composition of the Terfenol-D target (Fe: 66%, Tb: 10%, and Dy: 24%), and hence indicating that the average chemical composition of grown films is Fe_0.66_Dy_0.24_Tb_0.1_)_3_O_7-x_.

**Figure 2:**
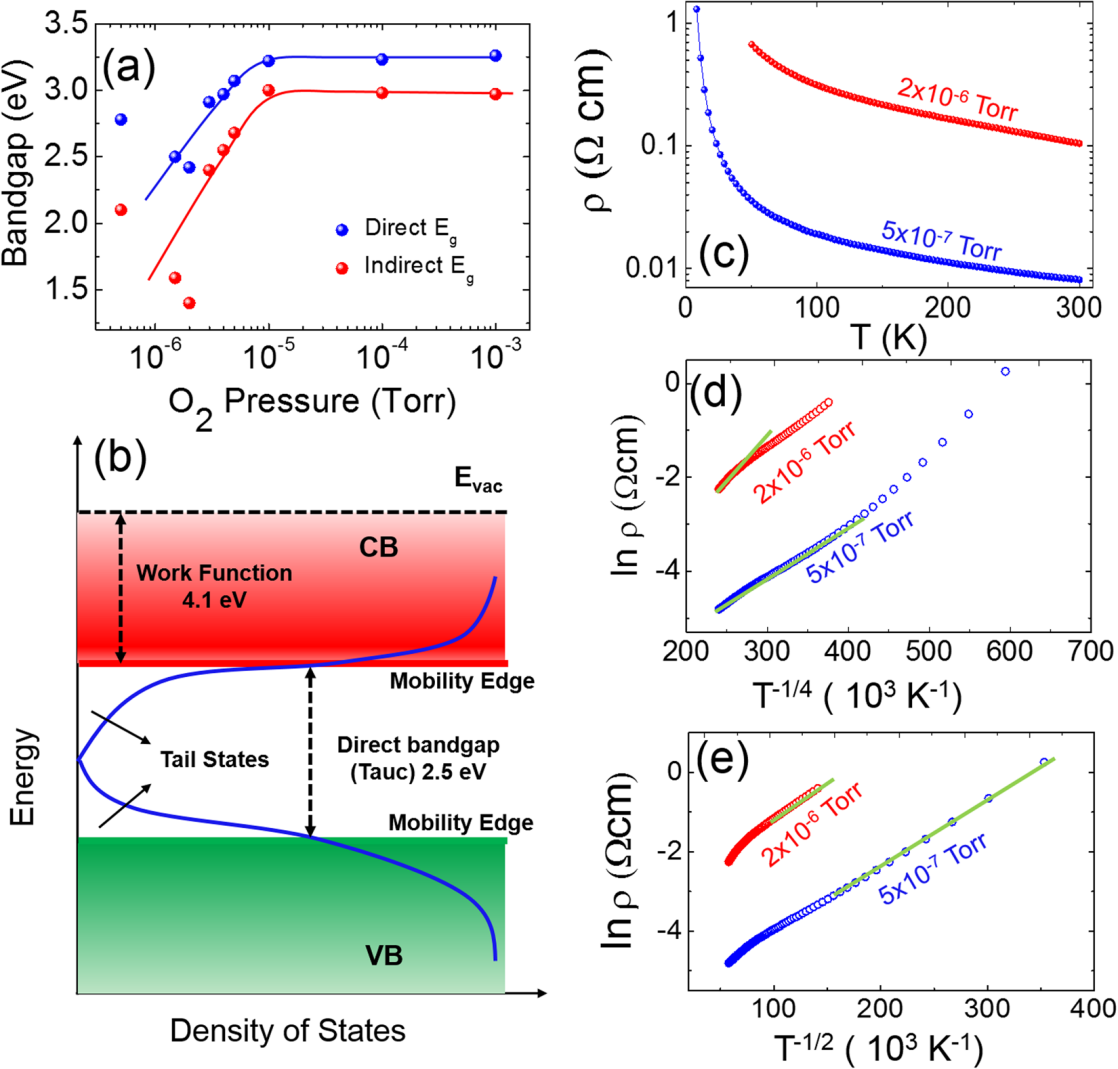
Band-gap and electrical conductivity of amorphous FDTO. (**a**) Direct and indirect bandgap obtained from Tauc plot for thin films of a-FDTO deposited at oxygen pressures ranging from 5 × 10^−7^ Torr to 1 × 10^−3^ Torr. (**b**) Schematic of band structure of a-FDTO film deposited at 2 × 10^−6^ Torr showing the bandgap, work function, and mobility edges and tail states at the conduction and valence bands. (**c**) Resistivity as a function of temperature from 3 K to 300 K for two a-FDTO films deposited at two different oxygen pressures. Both curves show semiconductor behavior. (**d**), (**e**) Fit of temperature dependent resistivity to (**d**) 3-D variable range hopping model, showing good fit for both a-FDTO films above 100 K, and (**e**) 1-D variable range hopping model showing good fit for both films below 100 K.

The electronic band gap of the FDTO thin films were investigated by a combination of optical and XPS techniques. In Fig. 2(a) the Tauc plot derived from optical transmission spectroscopy measurements (see Fig. S3, supplementary information) revealed that the films had a direct bandgap ranging from 2.5 eV (oxygen pressure 1 × 10^−6^ Torr) to 3.2 eV (oxygen pressure 1 × 10^−3^ Torr). The indirect bandgap was also seen to increase from 1.7 eV to 2.8 eV as pressure increased across the same range. The optical transmission measurements, shown in Supplementary Fig. S3(a) indicated that the films were optically transparent, although transparency decreased with decreasing oxygen pressure. To further elucidate the band structure, XPS measurements were used to measure the work function and valence band maximum (VBM) of the FDTO films deposited at 1 × 10^−6^ Torr and 5 × 10^−6^ Torr. While both films had an identical work function of 4.4 eV (see Fig. S3(d),(e), supplementary information), the Fermi level was found to lie 0.25 eV inside the valence band for the lower pressure film (1 × 10^−6^ Torr), while it was 0.34 eV above the valence band for the higher pressure film (5 × 10^−6^ Torr). Considering all the aforementioned pieces of information, along with prior understanding of band structure of amorphous semiconductor films, the band structure of amorphous FDTO can be envisioned as shown in Fig. 2(b). The important information here is that the band-gap suggested a semiconducting material whose energy gap could be tuned by changing the oxygen pressure during growth. In addition, the position of the valence band maximum with respect to the Fermi level indicated FDTO to be a p-type semiconductor. This band structure analysis suggests the interesting possibility that quantum-well homo-structures could be made in a monolithic film by simply tuning the oxygen pressure during growth.

The transport properties of the films were investigated as a function of temperature to determine the nature of electronic conduction. Figure 2(c) shows the temperature-dependent resistivity between 3–300 K for the lower oxygen pressure films. The films displayed decreasing resistivity with increasing temperature, indicative of semiconducting behavior. Films deposited at pressures of 1 × 10^−5^ Torr and higher show extremely high resistivity at room temperature (see Fig. S4(a), supplementary information), which did not change much with changing oxygen pressure, and thereby suggests that the films deposited at higher oxygen pressures are highly insulating in nature. The conduction in many disordered systems can be explained by either variable-range hopping (VRH) or small-polaron hopping (SPH)^39,40^. The magnitude of conductivity observed in the FDTO system for deposition pressures below 5 × 10^−6^ Torr, as shown in Fig. 2(c), is of the order of 10^−1^ to 10^2 −1^ cm^−1^ and is much higher than those typically observed for systems with SPH conduction (highest being 10^−3 −1^ cm^−1^ for vanadium oxide^41^), VRH seemed the more likely conduction mechanism. In 3-D VRH, the DC conductivity, is related to the temperature through a parameter, which can be expressed as 1/(d + 1), d being the space dimension of the system [38, 39] and 1/4 ≪ 1.0. The value of is largely determined by the localized DOS near the Fermi level, as explained by Singh and Shimakawa^42^. In case of a constant DOS, = 1/2 is obtained; however, the value of can be less than unity in the case of modified VRH if the fractal nature of the system is taken into account^39,43^. Therefore, selecting the correct model to apply is not trivial. In the case of FDTO, the most typical fit of 3-D VRH was applied to the low-temperature resistance data for both the films. As Fig. 2(d) shows, the experimental data fit very well with 3-D VRH (with T^−1/4^) down to 100 K, below which a 1-D VRH (T^−1/2^) fit the experimental data best, as shown in Fig. 2(e). The transition from 3D-VRH to 1D-VRH is a result of Coulomb interactions playing a greater role at low temperature where the kinetic energy of the electrons is also low.

**Figure 3:**
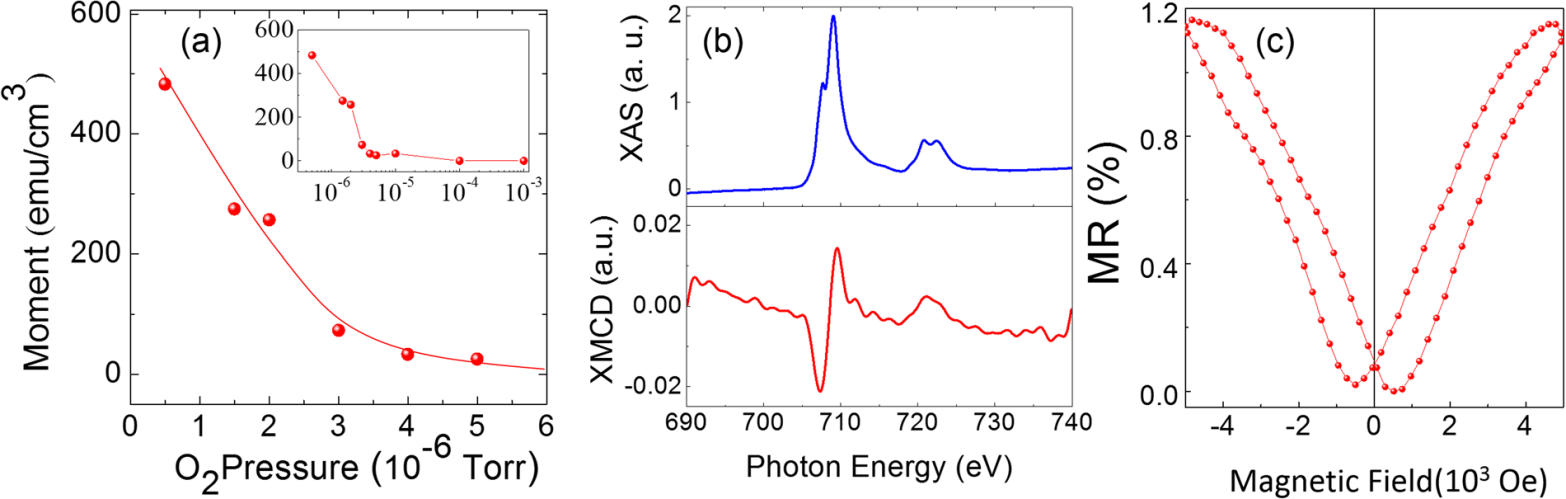
Magnetic properties of amorphous FDTO films. (**a**) Room temperature magnetic moment of FDTO films as a function of oxygen pressure during deposition. Inset shows the magnetic moment for a wider range of deposition pressure (up to 10^−3^ Torr) with a logarithmic x-axis. (**b**) XAS (top) and XMCD (bottom) data from an FDTO film deposited at 2 × 10^−6^ Torr showing the magnetic signal aligning with the absorption energy from Fe^3+^ peak. (**c**) Room temperature magnetoresistance of FDTO film deposited at 4 × 10^−6^ Torr showing positive magnetoresistance.

SQUID magnetometry of the FDTO films, deposited at varying oxygen pressure on quartz substrates, reveals a systematic variation of the saturation moment with the growth oxygen pressure, Fig. 3(a) (for details, see Fig. S4(b), supplementary information). For films deposited at oxygen pressures below 3 × 10^−6^ Torr the magnetic moment was >100 emu/cc at room temperature. The inset shows that those films prepared at oxygen pressures above 1 × 10^−5^ Torr exhibited no room-temperature remanent magnetism. Based on the presence of iron cation states in these films, X-ray magnetic circular dichroism (XMCD) measurements (Fig. 3(b)) were made on FDTO films deposited at 2 × 10^−6^ Torr to confirm that one of the sources of magnetism was the iron cation state and not metallic iron. The split peak appearing in the X-ray absorption spectroscopy data in the top panel of Fig. 3(b) is evidence of Fe^3+^ in the film, consistent with Fig. 1(d). X-ray measurements at the iron L_2,3_ edges revealed strong dichroism [Fig. 3(b), bottom panel], indicative of a net ferromagnetic moment, arising from the iron site. This result further supports the ferromagnetic state of the film, consistent with the SQUID results.

These magnetic, optical, and transport properties demonstrate that FDTO is an amorphous semiconductor displaying magnetic ordering at room temperature. However, in order for it to be useful in spintronic devices, the spin and charge carriers in the system must be coupled. One way to prove this is by confirming magnetoresistance in the system. Figure 3(c) shows the room-temperature magnetoresistance curve of an FDTO film deposited at 2 × 10^−6^ Torr, demonstrating positive magnetoresistance where resistance increases with increasing magnetic field, a phenomenon that has been reported in several disordered and granular magnetic systems^44–48^. Although the %MR in FDTO is lower than that in Fe_3_O_4_, the existence of magnetoresistance confirmed the coupling between spin and charge carriers in FDTO, and hence consolidating its utility towards magnetoelectric devices.

**Figure 4:**
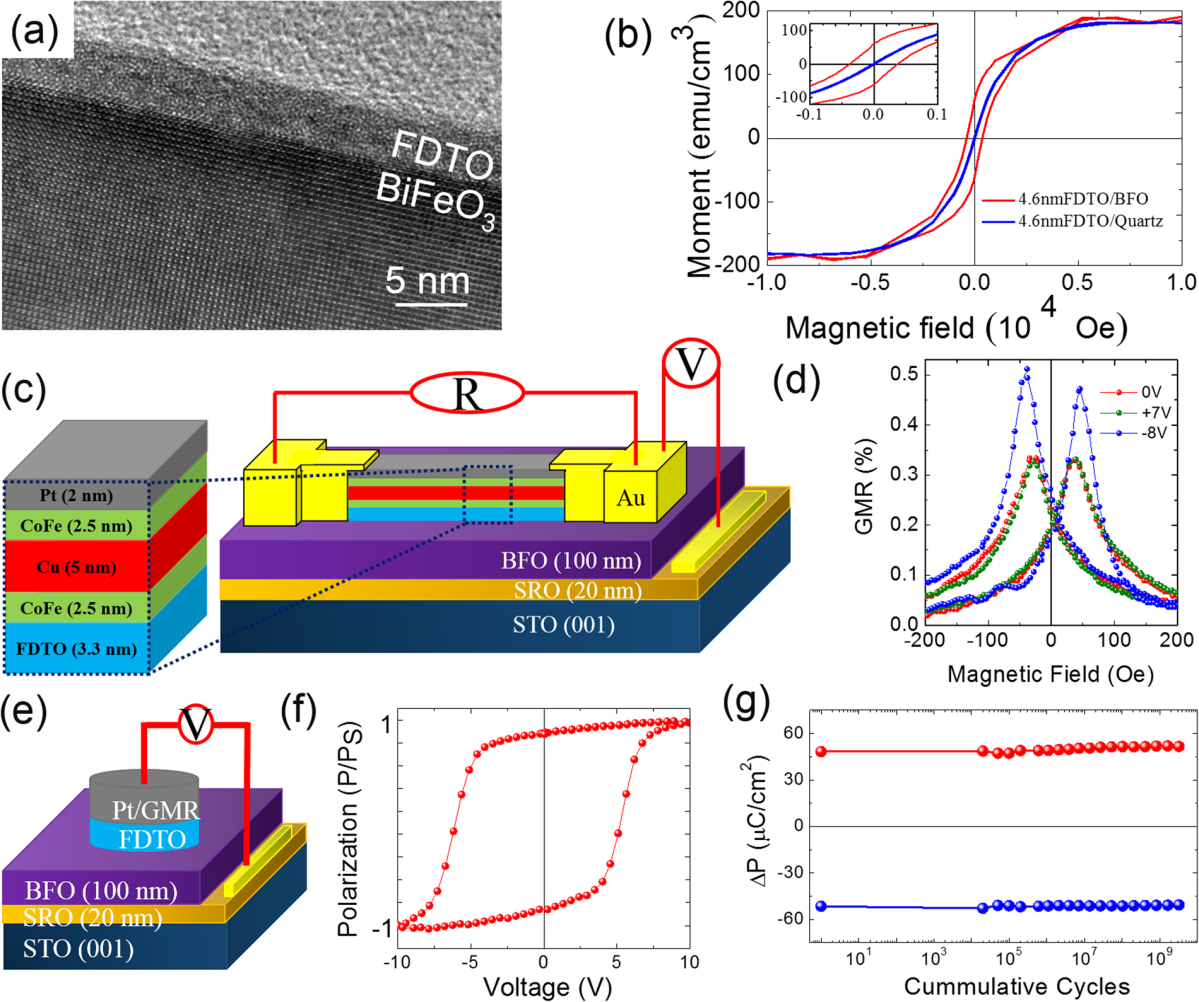
Magnetoelectric spintronics with ferromagnetic amorphous FDTO thin films. (**a**) HRTEM image of the interface between amorphous FDTO deposited at 2 × 10^−6^ Torr at room temeprature on crystalline BFO. (**b**) Magnetic moment (emu/cm^3^) loops taken at room temperature, showing enhanced coercivity of the FDTO/BFO film (red) as compared to the FDTO/quartz (blue), demonstrating magnetic coupling between FDTO and BFO thin film at room temperature. (**c**) Schematic of GMR heterostructure used to study ME switching capabilities of amorphous FDTO. (**d**) GMR signal as a function of applied magnetic field at room temperature showing change in the GMR signal upon switching BFO ferroelectric polarization from one state to another with the application of +7 V (green) to −8V (blue). (**e**) Schematic of a-FDTO/BFO capacitor used to test the robustness of FDTO layer as a top electrode for BFO. (**f**) Ferroelectric hysteresis loop of the capacitor in (**e**) at room temperature showing nearly rectangular closed hysteresis. (**g**) Fatigue test of the capacitor in (**e**) showing stable polarization for ~10^10^ cumulative switching cycles.

In order to examine the applicability of FDTO thin films for magnetoelectric devices, we studied the coupling to a known multiferroic, BiFeO_3_ (BFO). Figure 4(a) shows a cross-section high-resolution TEM image of amorphous FDTO film grown on single-crystal BFO/SrRuO_3_ at 2 × 10^−6^ Torr of oxygen pressure. The interface between FDTO and BFO was sharp and without any contamination, and the epitaxial oxide layers of BFO and SrRuO_3_ did not show any detrimental effect after the growth of FDTO films (see Fig. S5(a), supplementary information) The magnetic behavior of this system was compared to FDTO grown on quartz and is shown in Fig. 4(b). A substantial increase in coercivity for in-plane configuration, from ~10 to ~300 Oe, was seen for the FDTO on BFO compared to that on quartz. This strongly points to exchange coupling between the layers suggesting that the magnetic order in FDTO can couple to the underlying canted magnetic moments of BFO_._ Such a large enhancement in coercivity (observed in several samples, see Fig. S5(b), supplementary information) can be understood by the fact that the in-plane coercivity for a ferromagnetic metallic film (CoFe) changes, due to exchange coupling with BFO, from ~10 Oe to ~100 Oe^32,36^.

With this understanding of the magnetic coupling between the FDTO and BFO, the next step was to study the possibility of magnetoelectric coupling, i.e., electric field manipulation of the magnetic state of the FDTO. In order to do this, we have typically been using a spin valve as a sensing element^8,32^. Thus, as a first step, we fabricated a standard CoFe-Cu-CoFe giant magnetoresistance (GMR) heterostructure on the FDTO. Figure 4(c) shows the corresponding device schematic, where a bottom Co_0.9_Fe_0.1_ layer coupled to the BFO layer through the ferromagnetic FDTO, resulting in its higher coercivity compared to the top Co_0.9_Fe_0.1_ layer. This difference in coercivity between the top and the bottom Co_0.9_Fe_0.1_ layers gives rise to the GMR signal. Figure 4(d) shows a peak 0.35% GMR signal of the heterostructure when no voltage was applied across the BFO. Application of +7 V left the GMR signal unchanged, but application of −8 V markedly changed the GMR signal of the heterostructure. We have seen such a resistance modulation in several devices (see Fig. S5(c), supplementary information). The ferroelectric switching of BFO thin films with the amorphous FDTO as a top contact electrode was also tested and hysteresis loops were measured at room temperature on the capacitor device schematically shown in Fig. 4(e). The device showed stable polarization-electric field hysteresis loops as can be seen in Fig. 4(f), where the ferroelectric switching voltages match well with the magnetoelectric switching fields of spin valve devices fabricated on FDTO/BFO/SRO heterostructure (see Fig. 4d). In addition, bipolar polarization fatigue measurements showed at least ~10^10^ switching cycles, as seen in Fig. 4(g), a performance that is better than that for commonly used metallic electrodes such as platinum and comparable to the best crystalline oxide electrodes for BiFeO_3_^33–35^.

In conclusion, we have shown a new thin film oxide magnetic material (Fe_0.66_Dy_0.24_Tb_0.1_)_3_O_7-x_, that exhibits a desirable combination of magnetic moment (~200 emu/cc) and low enough resistivity (~8 mOhm-cm). The magnetism in the FDTO couples to the BiFeO_3_ at room temperature resulting in magnetoelectric switching that was probed through the fabrication of a simple spin valve structure. Also, FDTO could be used as a contact electrode and demonstrated stability with no indication of fatigue even after ~10^10^ ferroelectric switching cycles of BiFeO_3_. This work points to several directions for further research. An in-depth study of the degradation mechanisms would be extremely valuable. There has been very little use of amorphous metals as contacts to ferroelectrics and this work may be useful in that regard. Similarly, it would be of interest to create tunnel junctions using the FDTO as the magnetic layer (instead of the CoFe-Cu-CoFe on the FDTO), thus probing the spin transport directly.

## Methods

### Synthesis and fabrication

Thin films (20–30 nm) of FDTO were deposited from a commercially available metal alloy target, Terfenol-D (66% Fe, 24% Dy, 10% Tb) by pulsed laser deposition using a KrF excimer laser was used with wavelength of 248 nm, rep rate of 10 Hz, and energy density of 1 J/cm^2^. Quartz substrates were used to allow for transport, magnetic, and optical measurements on the films, as well as for GIXRD, XRR, TEM, and XPS. FDTO films were deposited at room temperature and in dynamic oxygen pressure environments of 5 × 10^−7^ Torr to 1 × 10^−3^ Torr. For the device studies, FDTO was grown as described above at 3 × 10^−6^ Torr on BFO (100 nm)/SRO (20 nm)/SRO (001) substrates in the presence of a magnetic field (~2500 Oe). The BFO and SRO were grown using the methods described in ref. ^32^ GMR structures of Co_0.9_Fe_0.1_ (2.5 nm)/Cu(5 nm)/Co_0.9_Fe_0.1_(2.5 nm) were deposited on FDTO(4 nm)/BFO(100 nm)/SRO(20 nm)/STO using magnetron sputtering techniques and subsequently the micron sized spin valves were created by conventional photolithography techniques. These same structures were used for ferroelectric measurements. The lateral dimension of the device was about 50 *μm*, while the device width was about 10 *μm*. Contacts were made by careful wire bonding to the Pt contact pads.

### Characterization techniques

#### Microstructure and chemical composition

- **X-ray reflectivity (XRR) and X-ray diffraction (XRD):** XRR was performed on the films Panalytical X’Pert^3^ MRD X-ray diffractometer in order to measure the thickness and density of the FDTO films. A slit of 1/16 was used on the beam side to optimize reflectivity signals. Fitting of the XRR fringes were done using the reflectivity software that came with the instrument. The same instrument was used to measure X-ray diffraction peaks of the BFO films.
- **Transmission electron microscopy (TEM):** The microstructure of the as-deposited and annealed FDTO films were further investigated in TEM to look for the presence/absence of any nanoclusters and the interfacial conditions. High-resolution TEM images, EELS spectra, and nano-diffraction patterns were taken in Ultra-STEM 200 microscope at the Center for Nanophase Materials Science at Oak Ridge National Lab, FEI Titan X 300KV microscope and F20 UT Tecnai 200KV at Lawrence Berkeley National Lab.
- **X-ray photoelectron spectroscopy (XPS)**: The as-prepared and annealed FDTO films were characterized by XPS to look at the cation states near the surface of the film. XPS measurements were done at room temperature in an ion-pumped chamber (evacuated to 2 × 10^−9^ Torr) of an PE-PHI5400 spectrometer, employing Al-Ka radiation (BE = 1486.6 eV) of about 4 mm spot size. The binding energy (BE) for the samples was calibrated by setting the measured BE of C 1 s to 284.6 eV. Peakfit software was further used to identify the chemical state of multifaceted Fe 2p, Dy Tb 4d and O1s spectra according to the previous reports. XPS measurements were also done to measure the work function of the as-prepared films. The system used for this purpose was a Kratos AXIS Ultra system which has a monochromatic Al K-alpha source, with a hemispherical analyzer. A charge correction to C 1s peak was applied during data fitting.

#### Optical properties

The optical characterization techniques utilized here were similar to those in our previous work^37^. Ultra-violet-Visible (UV-Vis) transmission spectra were obtained for all thin films deposited on quartz using an Ocean Optics spectrometer with a He-Ne light source that allowed measurements to be made between 300 nm to 900 nm, with integration time of 1 ms and 100 scans to average. Transmission spectra were acquired at five different locations of each sample to ensure homogeneity of the film thickness. Tauc plots for were generated by first converting the transmission values (% T) to absorbance using Beer-Lambert’s law and then dividing by the film thickness to get the absorption coefficient as a function of the probing wavelength. The Tauc plot was made with y-axis as ***(ahν)***^***1∕m***^ as a function of ***hν*** (the wavelength in energy units). A tangent was drawn at the region of the plot with sharp increase, which was then extrapolated to cut the x-axis at the band-gap value; m = 1/2 was used to obtain a direct band gap value and m = 2 was used to obtain indirect bandgap value assuming bands are parabolic.

#### Transport properties

Four-probe sheet resistance of the samples were measured at room temperature in the Van der Pauw geometry using Ecopia HMS 3000 Hall measurement system. Pt contact pads were deposited on the four corners of the samples prior to measurement. The samples were mounted on an SPCB-1 spring clipboard that comes with the HMS system. The contact probes were gold coated and spring loaded. Temperature and field dependent resistivity measurements were made using a Quantum Design Physical Property System. High purity silver epoxy from SPI Supplies and gold wires of 25 *μm* diameter were used as contact electrodes and wiring respectively. Measurements were made using the Van der Pauw configuration as described above to eliminate contact resistance at the sample-electrode interface, at intervals of 5 K.

#### Magnetic properties

Superconducting quantum interference device (SQUID) was used mainly to measure magnetic moment as a function of magnetic field. The system used was a Quantum Design Magnetic Property Measurement System using brass as a sample holder at UTK, and plastic drinking straw as sample holder at UCB. Magnetic fields of up to 5 Tesla were supplied for these measurements. Samples on quartz typically contributed to a diamagnetic background signal, which was subtracted to get the saturation magnetization for the FDTO films. Magnetic moment as a function of temperature were also measured for the as-prepared films having different R values using a vibrating sample magnetometer by our collaborator at North Carolina A&T State University. All samples for magnetic measurement were handled using non-magnetic, teflon tip tweezers to prevent contamination from magnetic impurities.

#### X-ray magnetic circular dichroism (XMCD)

XMCD measurements were carried out at beamline 6.3.1 of the Advanced Light Source, Lawrence Berkeley National Laboratory, focused on the Fe L-edge. The measurements used fixed circularly polarized X-rays at 300 K, in total electron yield configuration with grazing angle of 30°. To ensure that the XMCD signal was of magnetic origin, the measurements were repeated with opposite polarization and it was confirmed that the asymmetry reversed.

#### Magnetoresistance (MR)

- **Normal MR:** MR was measured using a home-built measurement system. The system used an electro-magnet that was capable of going up to 6000 Oe, a Keithley 6221 for supplying AC current, and a Stanford SR865A lock-in amplifier to measure the voltage. A LabVIEW program was developed to interface with all the instruments, and collect, plot and save data. The contacts were made by wirebonding Ag contact pads on the samples to contact pads on a chip holder that was used to mount the sample at the center of the magnetic field. The magnetic field was supplied IP to the plane of the sample for this measurement.
- **Giant MR (GMR):** GMR signal of the devices were measured using the same home-built system as described in the normal MR section. Magnetic field was supplied IP with respect to the plane of the sample. In addition, an Agilent signal generator was used to customize pulse voltages to be supplied as bias across the BFO thin film. A Keithley 6517a was also used for this purpose to supply a constant DC voltage when needed.

## Supplementary information

Supplementary information.
