# Supplementary material for: Integration of amorphous ferromagnetic oxides with multiferroic materials for room temperature magnetoelectric spintronics
Source: Sci Rep. 2020 Feb 27;10:3583. doi: 10.1038/s41598-020-58592-5 (PMC7046697; doi:10.1038/s41598-020-58592-5)
Supplement: Supplementary file 1 — Supplementary information. [file 41598_2020_58592_MOESM1_ESM.pdf]

Supplementary information

Integration of amorphous ferromagnetic oxides with multiferroic materials for room temperature magnetoelectric spintronics

Humaira Taz[1,2], Bhagwati Prasad[1], Yen-Lin Huang[1], Zuhuang Chen[1,3], Shang-Lin Hsu[1], Ruijuan Xu[1], Vishal Thakare[1], Tamil Selvan Sakthivel[4], Chenze Liu[5], Mark Hettick[6], Rupam Mukherjee[7], Sudipta Seal[8], Lane W. Martin[1,9], Ali Javey[6], Gerd Duscher[5], Ramamoorthy Ramesh[1]*, and Ramki Kalyanaraman[2,10]**

[1]*Department of Materials Science and Engineering, University of California, Berkeley, CA 94720, USA.*
[2]*Bredesen Center, University of Tennessee, Knoxville, TN 37996, USA*
[3]*School of Materials Science and Engineering, Harbin Institute of Technology, Shenzhen 518055, P. R. China*
[4]*Advanced Materials Processing and Analysis Center (AMPAC, Materials Science and Engineering (MSE) Department, University* of Central *Florida, Orlando, FL 32816, USA.*
[5]*Department of Materials Science and Engineering, University of Tennessee, Knoxville, TN 37996, USA.*
[6]*Department of Electrical Engineering and Computer Sciences, University of California, Berkeley, California 94720, USA.*
[7]*Department of Physics, Lovely Professional University, Phagwara, Punjab 144411, India.*
[8]*College of Medicine, University of Central Florida, Orlando, FL, 32827, USA.*
[9]*Materials Sciences Division, Lawrence Berkeley National Laboratory, Berkeley, California 94720, USA.*
[10]*Department of Chemical and Biomolecular Engineering, University of Tennessee, Knoxville, TN 37996, USA.*

Corresponding authors:
Prof. R. Ramesh, E-mail: rramesh@berkeley.edu
Prof. Prof. R. Kalyanaraman, E-mail: ramki@utk.edu
Dr. Bhagwati Prasad, E-mail: bprasadiitk@gmail.com

H.Taz and B. Prasad made equal contributions to the work.

Keywords: Amorphous oxide, Magnetic Semiconductor, Magnetoelectrics

Density and chemical composition:

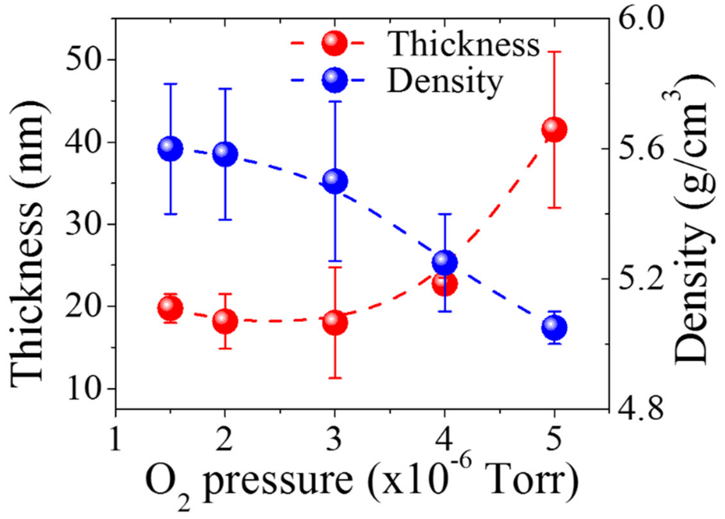

Figure S1. X-ray reflectivity (XRR) measurements of amorphous FDTO films for thickness and density analysis. Plots of density (blue, right y-axis) and thickness (red, left y-axis) of FDTO films, grown for same duration of time with same deposition conditions except the oxygen pressure, measured by XRR as a function of oxygen pressure, demonstrating the thicknesses of the a-FDTO films to be between 20 to 40 nm and the density to be between 5.1 and 5.6 g/cm^3 across the entire pressure range.

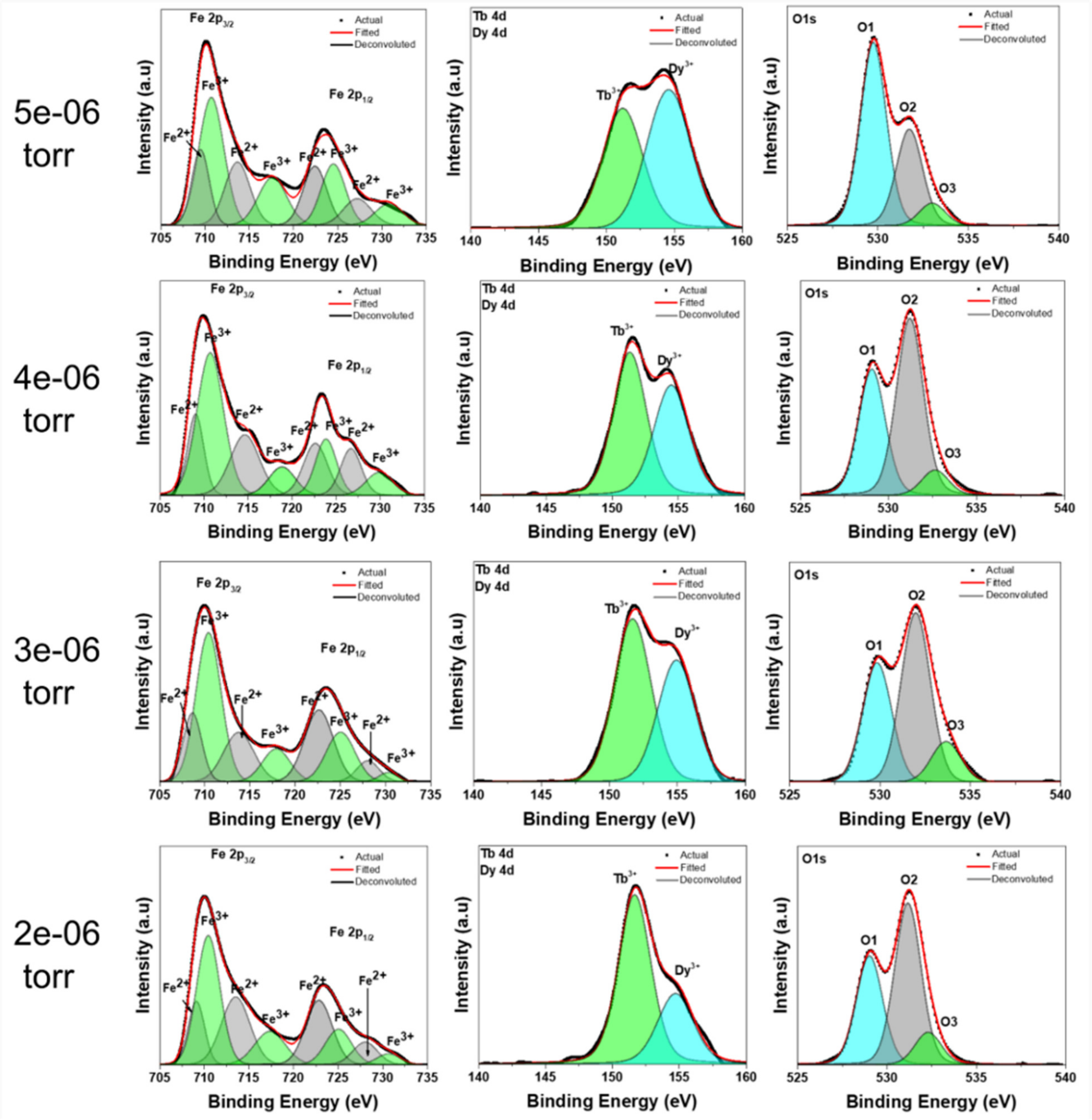

Figure S2. XPS measurements of the elemental cation states in amorphous FDTO. Figure panel showing XPS peaks obtained from FDTO films deposited at pressures from $5x10^{-6}$ Torr to $2x10^{-6}$ Torr. The columns represent Fe 2p, Tb and Dy 4d, and O 1s from left to right respectively, while the rows represent different oxygen pressures (labeled) from $5x10^{-6}$ Torr to $2x10^{-6}$ Torr from top to bottom. The spectra were fitted using Peakfit software (1), while the peak positions were obtained from the standard XPS database (2).

Optical properties and band gap:

Optical transmission measurements were performed on all the FDTO films to calculate their bandgap. Fig. S3(a) shows the optical transmission increasing for the FDTO films deposited at higher oxygen pressures. Tauc plot fits for both direct and indirect bandgaps (main plot and inset respectively), created from the transmission data, are shown in Fig. S3(b) and (c). From the x-intercept of the fits to the linear region of the Tauc plots, the bandgap values could be determined. The direct bandgap values were seen to start around 2.5 eV for the low-pressure samples and increase up to around 3.3 eV for the higher-pressure samples. Given that the bandgap value of Fe_2O_3, FeO, Tb_2O_3, and Dy_2O_3 are 2.2 eV (3,4), 3.5 eV (5), 3.8 eV and 4.9 eV (6), it can be concluded that the lanthanide oxides play an important role in determining the bandgap of the films as well. However, the work function of two FDTO films, one deposited at $5x10^{-6}$ Torr and another at $1x10^{-6}$ Torr, were the same value of 4.4 eV, as shown in Fig. S3(d). In contrast, their valence band maximum (VBM) edges were at different positions as shown in Fig. S3(e). The Fermi level was found to lie 0.25 eV inside the valence band for the lower pressure film, while it was 0.34 eV above the valence band for the higher-pressure film. This not only confirmed that both films were p-type semiconductors, but that at lower pressures, there was evidence for degenerate states. This could be the reason for the flatter shapes of the transmission curves observed in Fig. S3(a) for the lower pressure films when compared to the higher-pressure films.

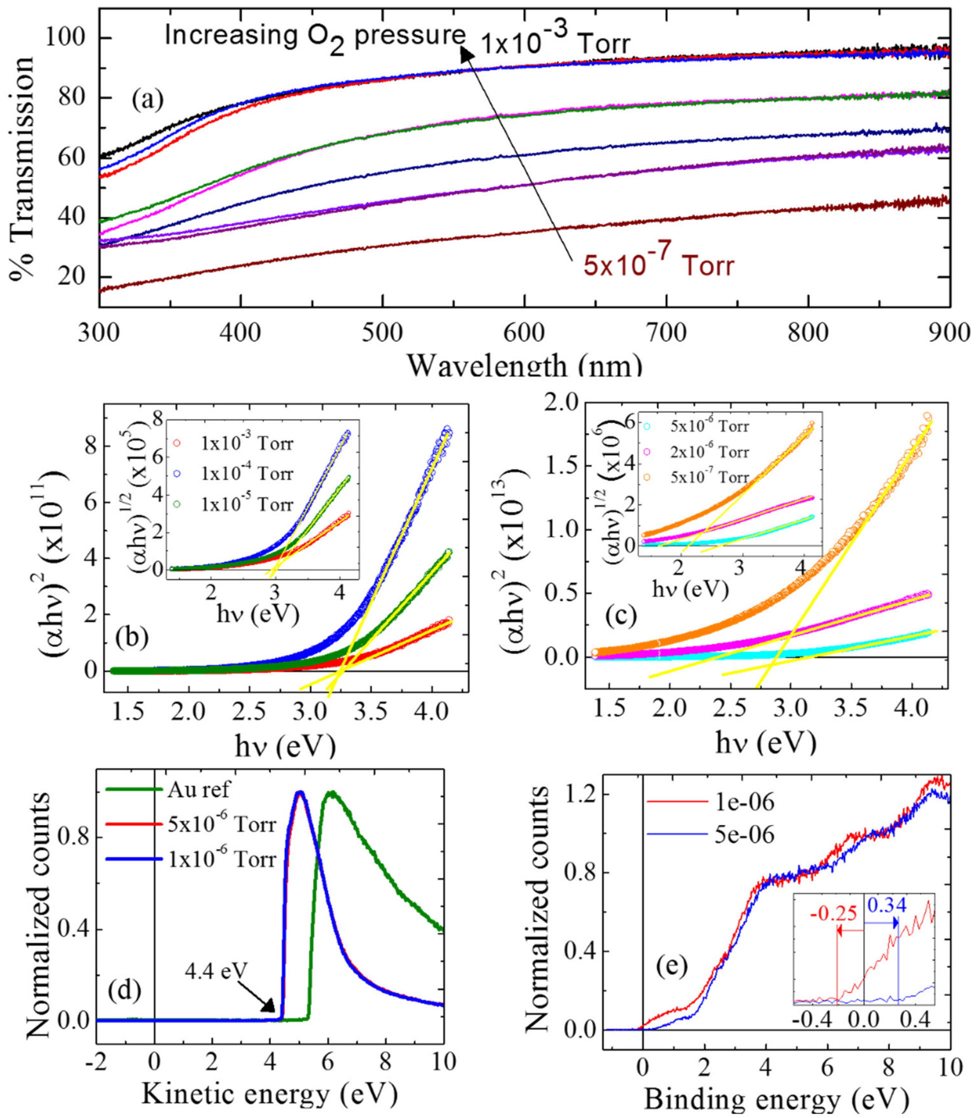

Figure S3. **Role of deposition oxygen background pressure on optical properties of amorphous FDTO films.** (a)Plot of optical transmission (%) for all FDTO films deposited at different oxygen pressures ($5x10^{-7}$, $1x10^{-6}$, $2x10^{-6}$, $3x10^{-6}$, $4x10^{-6}$, $5x10^{-6}$, $1x10^{-5}$, $1x10^{-4}$, $1x10^{-3}$ Torr), showing increase in % transmission as oxygen pressure increases. (b) (c) Tauc plot fits for direct bandgap of a-FDTO films at different oxygen pressures. Insets of (b) and (c) show the tauc plot fits for indirect bandgap for the same respective films. (d) Plot showing work function of FDTO films deposited at $5x10^{-6}$ Torr (red line) and at $1x10^{-6}$ Torr (green line); both films have the same work function of about 4.4 eV. (e) Valence band maximum edge obtained through XPS measurement of FDTO films deposited at $5x10^{-6}$ Torr (red line) and at $1x10^{-6}$ Torr (black line). The Fermi level is 0.25eV inside the valence band for the $1x10^{-6}$ Torr film, indicating degenerate states, while for the $5x10^{-6}$ Torr, it is 0.34 eV higher than the valence band edge. This indicates the films to be p-type semiconductors.

Resistivity and Magnetic properties of a-FDTO and its magnetic coupling to multiferroic $BiFeO_3$:

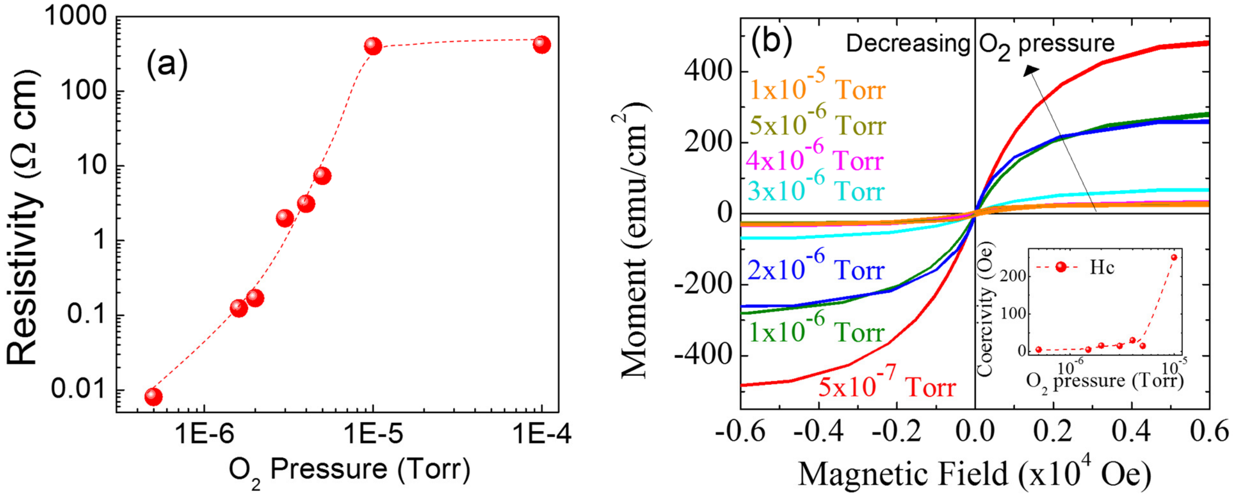

Figure S4. **Role of deposition oxygen background pressure on room temperature resistivity and magnetism of amorphous FDTO films.** Plot of (a) resistivity and (b) magnetic hysteresis loops for FDTO films deposited at different oxygen pressures; inset (b) plots the coercive field as a function of oxygen pressure, showing the coercivity to increase with increasing oxygen pressure.

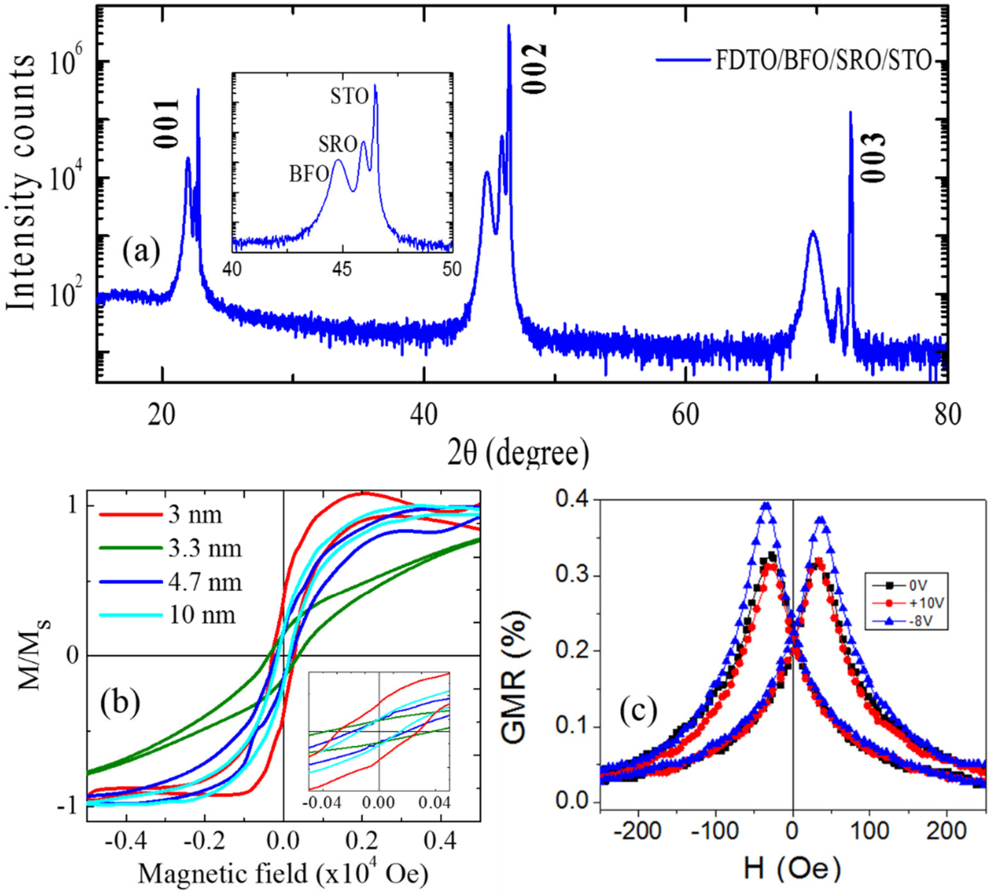

Figure S5. **Integration of FDTO thin films with BFO/SRO for magnetoelectric measurements.** (a) XRD pattern obtained at room temperature from FDTO/BFO/$SrRuO_3$/$SrTiO_3$ system, with the peaks consistent with the reported peak positions for the (001), (002), and (003) planes of BFO, $SrRuO_3$ and $SrTiO_3$, confirmed that the BFO crystal structure was not getting distorted due to the FDTO layer above it (7, 8). Inset shows a magnification of the (002) plane, showing the respective peak positions of BFO, $SrRuO_3$ and $SrTiO_3$. (b) Plot of room temperature magnetic hysteresis loops for different thicknesses of FDTO films on BFO, showing that the coercivity enhancement decreases as the thickness of FDTO is increased. This is evidence for exchange coupling between the FDTO and BFO layers. (c) GMR signal as a function of applied magnetic field of an additional device, measured at room temperature, showing change in the GMR signal upon switching BFO ferroelectric polarization from one state to another with the application of +10 V (red) to -8V (blue).

Supplementary Information References

1. http://www.sigmaplot.co.uk/products/peakfit/peakfit.php, URL http://www.sigmaplot.co.uk/products/peakfit/peakfit.php.

2. A.V. Naumkin, A. Kraut-Vass, S.W. Gaarenstroom, C.J. Powell, "NIST Standard Reference Database 20, Version 4.1". 2012.
URL: https://srdata.nist.gov/xps/elm_Spectra_query.aspx?Elm1=Tb&LD1=4d&Elm2=Tb&LD2=4d5.

3. Benjamin Klahr, Sixto Gimenez, Francisco Fabregat-Santiago, Juan Bisquert, Thomas W. Hamann, *En. Env. Sci.,* **2012**, 5, 7626.

4. Kevin Sivula, Florian Le Formal, Michael Gratzel, *Chem. Sus. Chem.*, **2011**, 4, 432.

5. W. H. Strehlow, E. L. Cook, "Compilation of Energy Band Gaps in Elemental and Binary Compound Semiconductors and Insulators", *J. Phys. Chem. Ref. Data*, **1973**, 2, 163.

6. G. Scarel, A. Svane, "Scientific and Technological Issues Related to Rare Earth Oxides: An Introduction" **2007**.

7. Liv R. Dedon, Sahar Saremi, Zuhuang Chen, Anoop R. Damodaran, Brent A. Apgar, Ran Gao, Lane W. Martin, "Nonstoichiometry, *Chem. Mat.*, **2016**, 28, 5952.

8. N. D. Scarisoreanu, F. Craciun, R. Birjega, V. Ion, V. S. Teodorescu, C. Ghica, R. Negrea, M. Dinescu, *Sci. Rep.,* **2016**, 6, 25535.
